# Detection of ripeness grades of berries using an electronic nose

**DOI:** 10.1002/fsn3.1788

**Published:** 2020-07-19

**Authors:** Nahid Aghilinategh, Mohammad Jafar Dalvand, Adieh Anvar

**Affiliations:** ^1^ Department of Agricultural Machinery Engineering Sonqor Agriculture Faculty Razi University Kermanshah Iran; ^2^ Faculty of Agricultural Engineering and Technology University of Tehran Karaj Iran; ^3^ Agricultural Science and Natural Resources University of Khuzestan Iran

**Keywords:** blackberry, electronic nose, ripeness grades, whiteberry

## Abstract

The estimation of ripeness is a significant section of quality determination since maturity at harvest can affect sensory and storage properties of fruits. A possible tactic for defining the grade of ripeness is sensing the aromatic volatiles released by fruit using electronic nose (e‐nose). For detection of the five ripeness grades of berries (whiteberry and blackberry), the e‐nose machine was designed and fabricated. Artificial neural networks (ANN), principal components analysis (PCA), and linear discriminant analysis (LDA) were applied for pattern recognition of array sensors. The best structure (10–11‐5) can classify the samples in five classes in ANN analysis with a precision of 100% and 88.3% for blackberry and whiteberry, respectively. Also, PCA analysis characterized 97% and 93% variance in the blackberry and whiteberry, respectively. The least correct classification for whiteberry was observed in the LDA method.

## Graphical abstract

According to our results, the e‐nose is a suitable instrument for detecting the ripeness grades of berries and can be used with less time and cost to determine the appropriate harvest time.

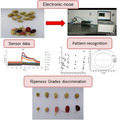

## 1 INTRODUCTION

Whiteberry (*Morus alba* L.) and blackberry (*Morus nigra* L.) (both known as mulberry) are deciduous trees belonging to the family Moraceae. Whiteberry and blackberry are the most important species of the *Morus* genus (Sánchez‐Salcedo, Mena, García‐Viguera, Martínez, & Hernández, 2015; Zelová et al., 2014). Mulberry fruits are highly appreciated by consumers for their aromatic taste. Mulberries provide nutrients and micronutrients essential for health and contain numerous chemical constituents, including tannins, phytosterols, polyphenolics, phytosterols, sitosterols, saponins, triterpenes, benzofuran derivatives, anthocyanins, glycosides, oleanolic acid, and volatile oils (Koyuncu, Çetinbas, & Erdal, 2014; Sánchez‐Salcedo et al., 2015).

The aroma is a complex composition of high volatile compounds, which are of tremendous great importance in food acceptability to consumers and a key indicator for evaluating fruit quality (Farrag, Kassem, Bayoumi, Shaker, & Afifi, 2017; Kim, Bae, Na, Dal Ko, & Chun, 2013). Due to the complex nature of the volatile profiles, the volatile composition is continuously changing in fruit. Many factors influence the volatile composition, including cultivar, fruit maturity, and postharvest environment (Forney, Kalt, & Jordan, 2000). Maturity is one of the critical factors influencing the abundance of volatile compounds in fruit (Lester, 2006). Ripening is a biochemical process in fruits, in which physical and chemical characteristics, including dramatic bioactive compounds production.

Controlling ripeness is becoming a fundamental issue in the fruit industry since ripeness during harvest, storage, and market distribution determines the quality of the final product measured in terms of customer satisfaction. An alternative strategy for determining the state of ripeness consists of sensing the aromatic volatiles emitted by fruit using e‐nose (Benady, Simon, Charles, & Miles, 1995; Hui et al., 2010; Kim et al., 2013; Llobet, Hines, Gardner, & Franco, 1999).

The e‐nose is one of the most promising nondestructive methods which have proven to be good alternatives for common techniques in odor analysis of food (Qiu, Wang, & Gao, 2015). The e‐nose contains several electronic gas sensors, which have sensitivity and selectivity to volatile compounds present in the sample headspace of food products. Through the use of a pattern recognition algorithm that processes the resistance data from each sensor, the volatile compound data are expressed as a thorough via multivariate analysis (Kim et al., 2013).

The statistical methods employed to multivariate output data obtained by the sensor array signals are based on commercial or specially designed software using multivariate classification methods like PCA, LDA, and ANN (Beghi, Buratti, Giovenzana, Benedetti, & Guidetti, 2017). The e‐nose has had several applications in monitoring aroma changes during fruits, such as apple (Pathange, Mallikarjunan, Marini, O’Keefe, & Vaughan, 2006), peach (Su et al., 2013; Zhang, Wang, Ye, & Chang, 2012), mango (Lebrun, Plotto, Goodner, Ducamp, & Baldwin, 2008; Zakaria et al., 2012), and tomato (Gómez, Hu, Wang, & Pereira, 2006).

Infante, Rubio, Meneses, and Contador (2011) applied an e‐nose for sensory quality evaluation of ripe nectarines segregated. The sensory analysis and the e‐nose results were presented through a PCA. Breijo, Guarrasi, Peris, Fillol, and Pinatti (2013) and Li, Xue, and Chen (2012) studied persimmon fruits to discriminate between two different cultivars using a semiconductor commercial e‐nose sensor array to recognize fruit ripening state and storage life, applying PCA and LDA statistical methods.

Zakaria et al. (2012) reported the classification of mango maturity levels using a fusion of the data of an electronic nose and an acoustic sensor. The e‐nose evaluated samples and then followed by the acoustic sensor. PCA and LDA were able to classify the mango harvested at week seven and week eight based solely on the aroma and volatile gases released from the mangoes. Parpinello et al. (2007) used the e‐nose to analyze the headspace of 10 different apricot cultivars. Applying a single hidden layer ANN with 35 neurons, a correlation index higher than 80% on test data set was achieved. Lu, Deng, Zhu, and Tian (2015) employed an e‐nose to classification of rice, and PCA was used to preprocess data from electronic systems.

This paper wants to investigate an application of e‐nose to detect the ripeness grade of berries, based on an e‐nose and proper pattern recognition methods (PCA, LDA, ANN), in whiteberry and blackberry.

## 2 MATERIALS AND METHODS

### 2.1 1. Electronic nose setup

**Figure 1:**
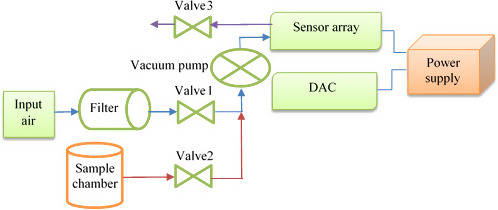
Schematic of the components of the electronic nose device

For detection of the ripeness of fruits, the e‐nose machine was designed and fabricated. The e‐nose mainly composed of data acquisition card (USB self‐designed), sensor array, three two‐way valves normally closed, vacuum pump, air filter (active carbon), GUI (graphical user interface) (LabVIEW 2014), power supply, laptop, and sample chamber. The schematic of the e‐nose apparatus is shown in Figure 1. The fruit is set in the sample chamber for collecting adequate gases.

In the e‐nose system, the static headspace technique is used to collect volatile (unstable) gases emitted from the sample. The vacuum pump with flow rate 6 L/min was used to transfer volatile and fresh air to the sensor array.

To automatically control the cleaning and obtain the aroma pattern, the e‐nose machine was equipped with three two‐way valves 1/4 inch normally closed. As the sample odor enters the sensor chamber, depending on the concentration of the sample violet, the type of sensor and sensibility of each sensor exhibit a proportional response that the sensor response converts into a voltage by its circuit and transmitted to the data acquisition card (USB self‐designed). This information is sent to the computer after being received by the data acquisition card for recording and display via the GUI (LabVIEW 2014). After each measurement, both chamber sample and sensor array are cleaned with filtered dry air by active carbon. The power required by the device is supplied in two ways, while the device is connected to the computer, required power is provided through the USB port of the computer, but when the e‐nose is used for on‐site experiments, the power (energy) required is supplied by the backup lithium‐ion battery.

The sensor array is combined with ten different metal oxide sensors. Table 1 shows all the sensors and corresponding applications. MOS gas sensors, as a typical commercial sensor, are extensively employed in e‐nose (Hu et al., 2016; Sun et al., 2017). These sensors are widely used in e‐nose machines due to their high chemical consistency, long life, low response to moisture, and reasonable prices (Sanaeifar, Mohtasebi, Ghasemi‐Varnamkhasti, & Ahmadi, 2016). Metal oxide semiconductor sensors need to be heated about 400°C before testing to detect the gases accurately. Therefore, sensors were equipped with a 5 V DC heating voltage. To omit the effects of temperature changes on the sensory array, the temperature of samples was held at 30°C.

**TABLE 1:** Gas sensor array of the e‐nose

Name	Main application	Detection ranges (ppm)
MQ3	Alcohol	0.05–10
MQ5	LPG, CH4, Coal gas	200–10,000
MQ9	CO and combustible gas	20–2,000 (Carbon monoxide), 500–10,000 (CH4), 500–10,000 (LPG)
MQ135	Air quality control	10–10,000 (Ammonia, Benzene, Hydrogen)
TGS2620	Alcohol, steam organic solvents	50–5,000
TGS2610	C4H10, LPG	500–10,000
TGS2611	CH4	500–10,000
TGS813	CH4, C3H8, C4H10	500–10,000
TGS822	Steam organic solvents	50–5,000
TGS2602	Sulfide, hydrogen sulfide, ammonia, toluene	1–30

The measurement procedure was started by placing fruits in the sample chamber. Preliminary experiments showed that the headspace achieved a steady state after the 1800s of equilibration, so those experiments were done after the 1800s of equilibration. They were designed to reinforce the odor concentration to obtain more sensor responses.

The main stages of electronic nose work consist of three phases: 1—baseline, 2—injection of sample odor into the sensor chamber, and 3—clearing the sensor array. The response of the sensors during these three‐time phases is recorded as voltage versus time.

In the baseline stage, the filtered air enters the sensor array by passing the vacuum pump and the valve 1 and cleans the sensor array to retain a stable voltage change in the sensors and was exited from valve 3 (300s). At the injection step, the sample odor enters the sensor array through the valve 2 and vacuum pump (300s). In the final stage, the filtered air enters the sensor array through the vacuum pump and valve 2, on the other hand, exits through the valve 3 to perform the cleanup process (150s) (Figure 1).

Data prepared from the sensors are applied to create a database required for training the e‐nose. Through the data attainment card, the sensors’ responses are saved on the computer. The database is a matrix whose rows are the responses of the sensors, and its columns are the e‐nose sensors. Then, signal preprocessing is used for the extraction of data from the obtained responses and also for the preparation of the data for pattern analysis (Wall, Rechtsteiner, & Rocha, 2003). The significant features of this preprocessing are (a) baseline identification, (b) compression, and (c) normalization.

The fractional method was employed in the current study for baseline manipulation. The fractional method is also extensively applied for MOS chemoresistors (Gutierrez‐Osuna, 2002)

$$y_{s}\left(t\right)=\frac{x_{s}\left(t\right)‐x_{s}\left(0\right)}{x_{s}\left(0\right)}$$

where *X_S_* (0) is the baseline response, *X_S_* (*t*) is the sensor response, and *Y_S_* (*t*) is the normalized response of the sensor. In compression preprocessing, the maximum response value for each sensor was individually extracted and analyzed. Using the fractional method in MOS sensors also, the data are normalized (Hai & Wang, 2006; Heidarbeigi et al., 2015).

### 2.2 Sample preparation

Whiteberry and blackberry samples were collected from 5‐year‐old mulberry trees from Iran. Healthy samples were randomly picked from multiple trees and divided into five ripeness grades (RG1 = ripe, RG2 = close to ripeness, RG3 = intermediate to ripeness, RG4 = close to unripe, and RG5 = unripe) according to the criteria used by expert growers (mainly relying on size and surface color distribution) during June 2019. The weight of each sample was measured as 10 ± 1 g. The 120 sample fruits were packaged in an insulated box containing ice and immediately transported to the laboratory for analysis.

### 2.3 Gas chromatography/mass spectrometry (GC/MS) analysis

#### 2.3.1 Sample preparation

A total of 100 g of the frozen mulberry fruit were ground in a commercial blender (Philips, model HR2850) for 30 s. The flesh pulp was then thawed for maceration at –4°C for two hours before centrifuged at 4000 × *g* at 4°C for 20 min. Finally, the sample was filtered through a muslin cloth to obtain the clean juice.

Five mililitre of the clean juice, 5 μl of 4‐methyl‐2‐pentanol (2.0200 g/L) as an internal standard, and 1 g of NaCl were added into a 15 ml vial, which was tightly capped with a polytetrafluoroethylene–silicon septum and sealed with a polypropylene screw cap. After mixing, the sample was equilibrated at 40°C on a magnetic platform (PC‐400, Supelco) for 30 min, a preconditioned 2 cm long 50/30 μm DVB/CAR/PDMS (divinylbenzene/carboxen/polydimethylsiloxane). SPME fiber (Supelco) was inserted through the cap and placed 1 cm above the juice to extract free volatiles at 40°C for 30 min. The SPME fiber was injected into a GC‐MS injector for thermal desorption at 250°C for 8 min. The same extraction procedure was previously employed for the aroma analysis of mulberry (Chen et al., 2015).

#### 2.3.2 GC‐MS analysis

An Agilent GC/MSD (7890A‐5975C) equipped with an HP‐INNOWax capillary column (60 m × 0.25 mm i.d. × 0.25 μm film thickness) from J&W Scientific (Folsom, Calif., U.S.A.) was used for the GC‐MS analysis. Helium was used as the carrier gas with a flow rate of 1 ml/min. The oven temperatures were programmed as follows: 50°C with a 1 min holding time, followed by an increase to 220°C at a rate of 3°C/min with a 5 min holding time. The HS SPME extract was injected in a splitless mode at 250°C. The temperatures of the detector and transfer line were maintained at 230 and 280°C, respectively. Mass spectra were acquired in electron impact (EI) mode at 70 eV with the *m/z* range of 40 to 250. Three replications of each sample were made in all cases.

### 2.4 Data analysis

One uncontrolled (PCA) and two controlled (ANN and LDA) pattern recognition models were used to classify fruit samples to varying degrees of ripening.

PCA is the best descriptor of differences between the samples. It has been mostly employed in the paper to display an embodiment of clusters and outliers of the e‐nose response to aroma (Li, Li, et al., 2012). Also, PCA helps to detect which principal components derived from the initial variances show the most differences.

LDA is one of the most used classification methods (Maugis, Celeux, & Martin‐Magniette, 2011). This technique minimizes the variance within categories and maximizes the variance between‐category differences (Patel, 2014). So, LDA can gather data from all sensors to amplify the groups.

In this research, a three‐layer feed forward neural network has been used that maps input data onto a set of proper outputs. In this research, the input layer of the network consisted of several neurons corresponding to sensors. The output layer had neurons according to grades of ripening fruits. Best number of neurons for the hidden layer were chosen basis on experiment and error. The data were divided into two subsets: 75% were applied for training, and 25% were used for testing. The hyperbolic tangent (tansig) and the linear (purelin) transfer functions were employed in the neurons of the hidden and output layers. The Levenberg–Marquardt training algorithm was used to train the network. Precision was applied as the classification performance function to find the optimal architecture for the neural network (Sokolova & Lapalme, 2009). For preparing the network, different numbers of neurons in the hidden layer were tested. In this work, an MSE of 10^−8^, a minimum gradient of 10^−10^ and a maximum epoch of 1000 were used. The primary weights and biases of the network were generated by using the netting function by the program. The values of the learning rate and momentum coefficient were 0.02 and 0.9, respectively. Neural network classification performance by the percentage of accuracy and precision of the confusion matrix was determined, using the following equations (Ayari, Mirzaee‐Ghaleh, Rabbani, & Heidarbeigi, 2018):

$$\text{Accuracy}=\frac{N_{\text{TP}}+N_{\text{TN}}}{N_{\text{TP}}+N_{\text{TN}}+N_{\text{FP}}+N_{\text{FN}}}$$

$$\text{Precision}=\frac{N_{\text{TP}}}{N_{\text{TP}}+N_{\text{FP}}}$$

where *N*_TP_, *N*_TN_, *N*_FP_, and *N*_FN_ are the number of samples that are classified as true positive, true negative, false positive, and false negative, respectively.

## 3 RESULT AND DISCUSSION

### 3.1 GC results

In this study, GC‐MS was used for the determination of different volatile in berries. The volatile aroma compounds found in whiteberry and blackberry (Table 2) can be grouped into six chemical groups: aldehydes, esters, alcohols, furanone, sulfide compounds, and terpenes. These results are in similar with most of the results obtained in other studies (Du, Kurnianta, McDaniel, Finn, & Qian, 2010).

**TABLE 2:** Identified volatile compounds of whiteberry and blackberry detected by GC‐MS (mg/kg)

Compounds	Blackberry	Whiteberry
Esters		
Methyl butanoate	0.22 ± 0.02	0.19 ± 0.01
Ethyl butanoate	1.2 ± 0.02	0.61 ± 0.01
Ethyl‐2‐methyl butanoate	0.71 ± 0.01	0.45 ± 0.01
Methyl hexanoate	0.02 ± 0.01	0.1 ± 0.01
Ethyl hexanoate	0.14 ± 0.02	0.11 ± 0.01
2‐Hexyl acetate	0.03 ± 0.01	0.01 ± 0.01
Ethyl acetate	0.1 ± 0.01	0.05 ± 0.01
Methyl salicylate	0.01 ± 0.01	0.02 ± 0.01
Butyl acetate	0.001 ± 0.06	ND
Isoamyl acetate	1.2 ± 0.03	0.67 ± 0.01
Hexyl butanoate	0.01 ± 0.01	0.03 ± 0.03
Octyl‐2‐methyl butanoate	ND	0.1 ± 0.01
Methyl acetate	0.33 ± 0.03	0.1 ± 0.03
Benzyl acetate	0.52 ± 0.02	0.32 ± 0.02
Hexyl hexanoate	0.12 ± 0.02	0.82 ± 0.01
Ethyl cinnamate	0.02 ± 0.02	ND
Pentyl Propyl hexanoate	ND	ND
Terpenoids		
Linalooll (3,7‐dimethylocta‐1,6‐dien‐3‐ol)	1.2 ± 0.03	0.42 ± 0.02
Nerolidol (3,7,11‐trimethyl‐1,6,10‐dodecatrien‐3‐ol)	0.10 ± 0.02	0.32 ± 0.02
α‐Terpineol	93 ± ± 0.01	86.01 ± 0.15
L‐α‐Terpinolene	13.9 ± 0.2	10.61 ± 0.12
Geraniol	30 ± ± 0.01	41.02 ± 0.02
trans‐Linalool oxide	30 ± ± 0.03	59.02 ± 0.02
Borneol	8.6 ± 0.8	5.02 ± 0.01
*l*‐carvone	3.8 ± 0.5	1.02 ± 0.02
Nerol	3.8 ± 0.3	1.8 ± 0.02
Nopol	2.8 ± 0.2	0.82 ± 0.02
Linalyl formate	ND	ND
Aldehydes		
Nonanal	ND	0.07 ± 0.04
(E)‐2‐nonenal	ND	0.08 ± 0.02
trans‐2‐Hexenol	0.32 ± 0.03	ND
Hexanal	0.06 ± 0.08	5.3 ± 0.02
cis‐3‐Hexenol	0.08 ± 0.05	0.09 ± 0.02
€‐2‐hexenal	3.08 ± 0.02	2.01 ± 0.03
Benzaldehyde	0.79 ± 0.01	0.35 ± 0.02
Alcohols		
1‐Octanol	0.08 ± 0.02	0.04 ± 0.02
2‐Heptanol	0.35 ± 0.05	0.23 ± 0.05
1‐Octen‐3‐ol	1.1 ± 0.03	0. 5 ± 0.02
Benzyl alcohol	1.9 ± 0.05	0.95 ± 0.02
Phenethyl alcohol	0.06 ± 0.05	0.25 ± 0.05
Sulfide compounds		
Hydrogen sulfide	0.03 ± 0.01	0.05 ± 0.01
Methanethiol		0.08 ± 0.02
Dimethyl sulfide	0.02 ± 0.01	0.08 ± 0.02
Dimethyl disulfide	0.01 ± 0.001	ND
Dimethyl trisulfide	0.01 ± 0.001	ND
Methyl thioacetate	ND	ND
Methyl thiobutyrate	ND	0. 01 ± 0.001
Acids		
2‐Methylbutanoic acid	0.05 ± 0.01	0.02 ± 0.01
Hexanoic acid	0.75 ± 0.05	0.09 ± 0.03
Acetic	0.72 ± 0.01	0.63 ± 0.01
Nonanoic	ND	ND
2‐Methyl‐3‐hydropropanoic	ND	0.21 ± 0.02
Furanone		
Mesifurane	0.13 ± 0.01	0.85 ± 0.01
Furaneol	1.65 ± 0.01	2.01 ± 0.01
Lactones		
*γ‐*Decalactone	2.5 ± 0.05	3.35 ± 0.01
*γ‐*Dodecalactone	2.05 ± 0.01	1.05 ± 0.01
*γ*‐Octalactone	3.8 ± 0.1	ND
γ‐Nonalactone	ND	0.06 ± 0.02
*γ*‐Undecalactone	1.3 ± 0.03	0.6 ± 0.02
sigma‐Decalactone	1.4 ± 0.2	0.6 ± 0.02
Others		
2‐Heptanone	3.3 ± 0.01	1.8 ± 0.04
Eugenol	3.8 ± 0.5	2.8 ± 0.01
Isoeugenol	5.4 ± 0.01	7.8 ± 0.06

ND Not detected

More than 40 aroma compounds have been identified in blackberry, while around 30 aroma compounds have been found in whiteberry. Alcohols and esters have been described as the major aroma compounds of the Morus genus. The concentration of these volatile compounds is generally low, and they can be affected by several agronomic such as ripening stage and technological factors (Calín‐Sánchez et al., 2013).

### 3.2 Comparison of sensor array responses and GC results

**Figure 2:**
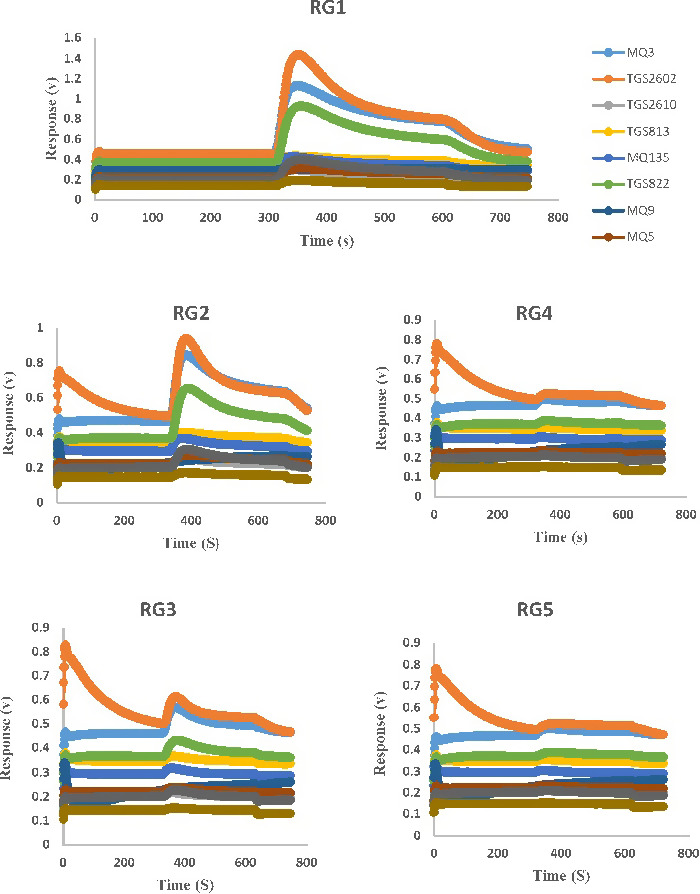
Whiteberries grouped in five ripening grades (RG1 = ripe, RG2 = close to ripeness, RG3 = intermediate to ripeness, RG4 = close to unripe, and RG5 = unripe)

The response of the sensory array for whiteberry is shown in Figure 2. As it is known, RGs have different response patterns. This is due to change in volatile aroma compounds during maturation.

Many rapid qualitative and quantitative changes in berry fruit volatiles during ripening is related to steam organic solvents such as esters, furaneol and mesifurane, acids, lactones, terpenes, and alcohols (Forney et al., 2000; Jetti, Yang, Kurnianta, Finn, & Qian, 2007; Ménager, Jost, & Aubert, 2004).These important volatile compounds were detectable by MQ3 and TGS822 sensors as well as GC‐MS (Table 1 and Table 2). Volatile sulfur compounds can be presented in many different chemical forms, including hydrogen sulfide, methanethiol, dimethyl sulfide, dimethyl disulfide, dimethyl trisulfide, methyl thioacetate, and methyl thiobutyrate that have been identified by GC‐MS (Table 2) and TGS2602 sensors (Table 1) in whiteberry and blackberry fruits.

The harvest maturity plays a pivotal role in the volatile development of berries. C6 aldehydes were identified as the major compounds in immature white fruit, while furanone and esters are present in three quarters or fully red fruit (Ménager et al., 2004).

Yang, Wang, Wu, Fang, and Li (2011) reported that all the organic compounds such as alcohols and carbonyls, along with most of the C6 compounds and terpenoids, were evident before veraison in three different flavor table‐grapes, while most of the esters were detected at or after veraison. C6 compounds increased in the early period of maturation and then decreased. Most alcohols and carbonyls tended to decrease during ripening continuously. Some esters continued to increase after maturation and, terpenoids increased until maturation, then reduced. Du, Song, and Rouseff, (2011) reported with increasing degree of maturity, volatile sulfur concentrations increased and at full ripe and overripe maturity stages increased exponentially.

### 3.3 PCA results

**Figure 3:**
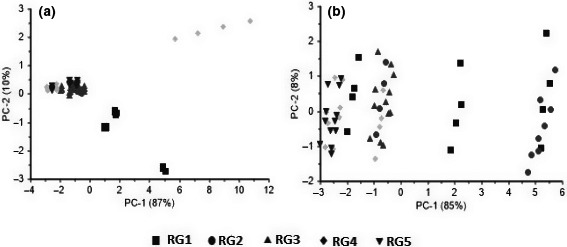
Score plot of PCA analysis for detection ripeness of (a) blackberry and (b) whiteberry

The first two main components of PCA showed more than 90% of the variance of data; therefore, these two components were used for PCA plots (Figure 3). The PCA score plots of PC1–PC2 explained 97% and 93% of the variance for blackberry and whiteberry, respectively.

In Figure 3a, PC1 and PC2 described 87% and 10%, respectively, of the variance between samples. All of the RG were clustered well by PCA (Figure 3a). In Figure 3b, PC1 and PC2 described 85% and 8%, respectively, of the variance between samples. RG4 and RG5 and RG1 and RG2 overlapped.

To determine the contribution of each sensor in the pattern recognition analysis, a loading plot was used. The sensors are displayed in the loading plot with specific coefficient values. The high coefficient value for a sensor in the loading plot indicates the important role of this sensor in detecting RGs of berries. Also, by eliminating the sensors that play the least role in detecting RGs, in addition to reducing the complexity of the data analysis process, the cost of the fabricated array of sensors is reduced.

**Figure 4:**
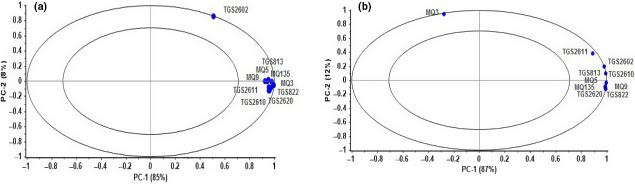
Loading plot of PCA analysis for detection ripeness of (a) blackberry and (b) whiteberry

In the case of whiteberry, all sensors gave an excellent contribution. However, MQ9 and TGS2611 have the lowest response than to other sensors in whiteberry (Figure 4b). According to Figure 4a, sensors MQ3 and TG2602 showed the highest contribution, and sensors MQ9 and TGS2610 had the lowest response. Calín‐Sánchez et al. (2013) reported that black and white mulberry species displayed significantly different characteristics.

In a research conducted by Pokhum, Chawengkijwanich, and Maolanon (2009), the use of e‐nose for identification of the ripeness stage of durian was investigated. PCA technique was applied for data analysis. The PCA result showed that volatile durian profile at unripe, ripe, and overripe stages was significantly classified. The results of PCA characterized a clear distribution in the groups; it cited that the selected sensors can reverberate the difference in volatile compound released from samples.

### 3.4 LDA results

**Figure 5:**
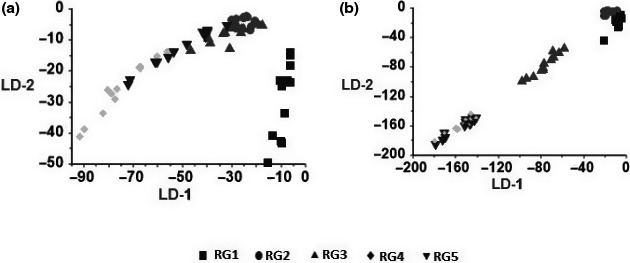
LDA analysis for detection ripeness of (a) blackberry and (b) whiteberry

For blackberry, LDA could distinguish RGs well, but RG4 and RG5 and RG2 and RG3 have little overlap. The accuracy of the analysis was 96.67% (Figure 5a). LDA was not able to identify RGs of whiteberry well, because RG4 and RG5 and RG1 and RG2 have a great overlap. The accuracy of the analysis was 85% (Figure 5b). In two berries was seen overlap between RG4 and RG5; therefore, it can be concluded that there is no clear difference in the aromatic compounds of these two groups especially in whiteberry.

Qiu et al. (2015) were studied about discrimination and characterization of strawberry juice based on electronic nose and tongue. LDA, PLSR, RF, and SVM methods were applied for data analysis. The result showed LDA is useful tool for discrimination.

### 3.5 ANN results

To minimize ANN training time, only one hidden layer was considered. The best network was found with 10–11‐5 topology, that is, a network having 11 neurons in the hidden layer for all berries. Table 3 shows the confusion matrices. Samples were classified with correct classification percentage of 88.3% and 100% for whiteberry and blackberry. The lowest classification and precision were seen for RG4 and RG5 of whiteberry which can be due to the slight difference in violate compounds. Most fruit flavor volatiles are secondary metabolites and absent during the early grades of fruit formation. Therefore, fruits in first grades of maturity are not separated and could not be differentiated using volatile compounds. These results are in agreement with the results of LDA.

**TABLE 3:** Confusion matrix obtained to five ripeness of berries

	1	2	3	4	5	Precision	Accuracy	Correct classification percentage
Blackberry								
1	12	0	0	0	0	100	100	100%
2	0	12	0	0	0	100	100	
3	0	0	12	0	0	100	100	
4	0	0	0	12	0	100	100	
5	0	0	0	0	12	100	100	
Whiteberry								
1	12	0		0	0	100	99.8	88.3%
2	0	12	0	0	0	100	96.22	
3	0	0	12	0	0	100	95.8	
4	0	0	0	8	4	66.6	85.54	
5	0	0	0	3	9	75	87.6	

The results of Du et al. (2011) indicated that volatile sulfur concentrations were mostly absent at the early maturity grades, such as the white and red half grades.

Brezmes, Llobet, Vilanova, Saiz, and Correig (2000) investigated fruit ripeness monitoring using an e‐nose. Based on the neural network as a pattern recognition technique, the system designed was able to categorize fruit samples into three different grades of ripeness green, ripe, and overripe with prefect accuracy.

## 4 CONCLUSION

In this research, a fabricated electronic nose with ten metal oxide semiconductor sensors with LDA, PCA, and ANN to determine the ripeness grades of whiteberry and blackberry was used.

Three pattern recognitions were able to classify the RGs of berries well. But ANN and PCA for blackberry with the correct classification percentage 100% and explanation 97% of the variance of samples are the best methods. According to the study, it can be expressed that an e‐nose is a useful tool for detecting the ripeness grades of berries and can be used with less time and cost to determine the appropriate harvest time. MQ3 and TGS2602 sensors showed the highest contribution and MQ9, TGS2611, and TGS2610 sensors showed the lowest response in identifying the RGs of berries.
